# Draft Genome Sequence of the Soil Isolate *Lysinibacillus fusiformis* M5, a Potential Hypoxanthine Producer

**DOI:** 10.1128/genomeA.01272-16

**Published:** 2016-11-10

**Authors:** Ramses Gallegos-Monterrosa, Gergely Maróti, Balázs Bálint, Ákos T. Kovács

**Affiliations:** a Terrestrial Biofilms Group, Institute of Microbiology, Friedrich Schiller University Jena, Jena, Germany; b Institute of Biochemistry, Biological Research Centre, Hungarian Academy of Sciences, Szeged, Hungary; c Seqomics Biotechnology Ltd., Mórahalom, Hungary

## Abstract

*Lysinibacillus fusiformis* strain M5 is a potential hypoxanthine producer that was isolated from clay soil. Here, we present the draft genome sequence that was annotated in order to facilitate future studies of *L. fusiformis* M5.

## GENOME ANNOUNCEMENT

*Lysinibacillus fusiformis* is a Gram-positive endospore-forming soil bacterium that was recently reclassified from the *Bacillus* genus due to differences in its cell wall components (1). Although *L. fusiformis* has been suspected for it pathogenicity (2–4), other studies reported the isolation of this species from diverse environmental samples, and it has been proposed as a potential producer of industrially attractive metabolites (5, 6).

Screening of a library of isolates obtained from a Mexican clay soil collected at the warm and humid region of Tepoztlán, Morelos, resulted in the identification of *L. fusiformis* M5. It was selected for further study due to its ability to produce hypoxanthine (R. Gallegos-Monterrosa and Á. T. Kovács, unpublished data). Hypoxanthine is a common metabolite produced by bacteria as part of the purine salvage pathway (7, 8). This nucleobase and its concomitant enzymes have been extensively studied due to their role in cell metabolism and signaling, and as potential drug targets (9, 10).

We performed whole-genome sequencing of *L. fusiformis* strain M5 in order to facilitate the identification of genes involved in hypoxanthine production. Genomic DNA of *L. fusiformis* M5 was isolated with GeneMATRIX bacterial and yeast genomic DNA purification kit, according to the manufacturer’s instructions (EURx, Gdańsk, Poland). A mate-pair library was generated using the Illumina Nextera mate-pair kit (catalog no. FC-132-1001), with insert sizes ranging between 7 and 11 kb. DNA sequencing was carried out on an Illumina MiSeq machine using V2 sequencing chemistry, resulting in 2 × 250-bp reads. Raw data were preprocessed for *de novo* assembly according to the manufacturer’s recommendations. Data processing of Nextera mate pair reads was performed using Illumina Sequencing Platforms (http://www.illumina.com/documents/products/technotes/technote_nextera_matepair_data_processing.pdf).

*De novo* assembly was performed with CLC Genomics Workbench 8.0.2 (CLC bio), with contigs being subsequently arranged into scaffolds using SSPACE 3.0 (11). Gaps in scaffolds were closed with SPAdes version 3.1.1 (12), together with an in-house R script (B. Bálint, unpublished data). The assembly produced 7 contigs and a circularized plasmid that comprise 4,744,577 and 134,678 bases, respectively, with G+C contents of 37 and 36%, respectively. Automated annotation was performed using the NCBI Prokaryotic Genome Annotation Pipeline (13); 4,753 genes were identified, including 74 tRNA and 22 rRNA regions. Around 96% of the identified genes corresponded to hypothetical proteins (4577 coding open reading frames [ORFs]).

Genes coding for proteins possibly involved in hypoxanthine production were identified among the annotated genes, namely, *pbuE*, a putative hypoxanthine transporter; and *adeC* and *yerA*, putative adenine deaminases involved in the purine salvage pathway. Genome comparison confirmed the presence of homologous genes (identity, ≥95%) in the genomes of *L. fusiformis* RB-21 (GenBank accession no. CP010820.1) and *L. fusiformis* SW-B9 (GenBank accession no. JRBA00000000.1) (14). Based on genomic BLAST, *L. fusiformis* M5 shows closest homology to *L. fusiformis* strain H1k (GenBank accession no. AYMK00000000.1).

### Accession number(s).

This whole-genome shotgun project has been deposited in GenBank under the accession no. MECQ00000000. The version described in this paper is the first version, MECQ00000000.1.
